# Minimally Invasive Surgical Versus Catheter Ablation for the Long-Lasting Persistent Atrial Fibrillation

**DOI:** 10.1371/journal.pone.0022122

**Published:** 2011-07-13

**Authors:** Jiangang Wang, Yan Li, Jiahai Shi, Jie Han, Chunlei Xu, Changsheng Ma, Xu Meng

**Affiliations:** 1 Department of Atrial Fibrillation Center, Beijing Anzhen Hospital, Capital Medical University, Beijing, China; 2 Department of Cardiothoracic Surgery, Affiliated Hospital of Nantong University, Nantong, China; University of Chicago, United States of America

## Abstract

**Objective:**

To assess the efficacy of video-assisted minimally invasive surgical vs. catheter ablation for the long-standing persistent AF.

**Methods:**

We performed a retrospective comparative analysis in a series of 166 long-standing persistent AF patients treated between 2006 and 2009 with either video-assisted minimally invasive ablation (83 patients), or catheter ablations (83 patients). The catheter group was screened using a ‘pair-matched case-control’ methodology in order to select appropriate statistical comparison candidates out of 169 long-standing persistent AF patients which were potentially suitable for surgery, but have been treated with catheter approaches in order to balance major prognostic factors between the two groups. Follow-up for all patients ranged from 1 to 3.6 years.

**Results:**

No patient died postoperatively. One patient suffered from stroke in the surgical group but recovered before discharge. Freedom from AF was obtained in 59.0% and 74.7% during follow-up in the catheter group and the surgical group respectively (*P* = 0.047). Patients in the catheter group had a significantly higher rate of recurrent arrhythmia (*P* = 0.011, hazard ratio: 0.555, 95% CI: 0.354 to 0.872) compared with the surgically treated group. The freedom from antiarrhythmic drugs was 44.6% in the catheter group and 61.4% in the surgical group (*P* = 0.043).

**Conclusions:**

The video-assisted minimally invasive ablation was safe and effective, and had an optimistic success rate for patients with long-standing persistent AF in our retrospective comparative study. Thus, further randomized studies addressing this issue seem to be justified.

## Introduction

Atrial fibrillation (AF) affects proximately 10 million patients in China, undoubtedly the largest demographic group in the world [1]. Catheter ablation targeting pulmonary vein isolation (PVI) has been evolved over the past decade and has become the common treatment for AF [2], but it often requires repeated procedures, and is associated with serious, although infrequent, complications [3], [4]. It has limited success in persistent and permanent AF, although different ablation strategies beyond PVI have been described to improve the outcome in this patient group [5], [6]. Wolf and colleagues reported encouraging early experiences with a stand-alone, minimally invasive, video-assisted thoracoscopic surgical technique for the epicardial ablation of AF on a beating heart with the use of dry radiofrequency (RF) bipolar energy [7].

To assess the clinical outcome of surgical ablation we performed a retrospective analysis comparing two pair-matched groups of patients with long-standing persistent AF, one treated with video-assisted minimally invasive ablation, and the other one treated with conventional catheter approach.

## Methods

### 2.1 Objective

In this restrospective study we compared the surgical minimally invasive surgery method versus the internal medicine catheter ablation method in order to evaluate an optimal proceeding for the treatment of long-standing atrial fibrillation and we hypothesised that minimally invasive surgery is the superior approach.

### 2.2 Patients

Between 2006 and 2009, 91 consecutive patients with long-standing persistent AF were treated with video-assisted thoracoscopic surgical ablation at the Atrial Fibrillation Centre, Beijing Anzhen Hospital. Long-standing persistent AF was defined as continuous AF lasting longer than 1 year, resistant to either electrical or pharmacological cardioversion. Indications for surgical AF ablation were: Drug-refractory; inability to tolerate antiarrhythmic drugs or anticoagulation therapy as well as left ventricular ejection fraction of 30% or greater. The patients should also have been able to provide a written informed consent, their life expectancy should have been at least 2 years and they must have been able to attend scheduled follow-up visits. The exclusion criteria in the surgical group were: left ventricular ejection fraction lower than 30%, sick sinus syndrome, severe pleural adhesions as well as prior attempts with catheter ablation for a treatment of atrial fibrillation, what means that none of the patients in the surgical group, which we have chosen for the comparison, underwent a previous catheter ablation. In order to assess the role of surgery in the treatment of long-standing persistent AF, treatment outcome in this group of patients was compared with that of patients managed with conventional catheter approaches. The inclusion criteria for a treatment with catheter AF ablation were: Symptomatic AF refractory or intolerance to at least one class 1 or 3 antiarrhythmic medication and patients with heart failure and/or reduced ejection fraction. In addition in rare clinical situations, it may be appropriate to perform catheter AF ablation as the first line therapy. The exclusion criteria in the catheter group were: Left ventricular ejection fraction <30 percent, presence of left atrial thrombus on transesophageal echocardiography and prior attempts with catheter or surgical ablation for an atrial fibrillation treatment.

To secure a relatively reliable comparative analysis between patients treated with and without surgery, we performed a matched case-control procedure. From the group of patients treated with catheter approaches we selected cases with similar favorable prognostic factors matching those in the surgical group and from a total of 169 patients treated with catheter approaches, we selected 89 cases which also would have been potentially suitable for surgery. Each patient from the surgical group has then been pair-matched to a patient from the screened catheter group, taking into account the most important prognostic factors, which were duration of AF, left atrial dimension and sex. Patients from both groups for whom no comparable control case could be found were then excluded from further analysis. As a result of this process, 166 patients were selected for a comparative analysis; 83 treated with minimal invasive surgery versus 83 managed with catheter approach (Table 1).

**Table 1:** Patient characteristics.

	Catheter GroupN = 83	Surgical GroupN = 83	*P* Value
Age (yrs)	55±12	57±11	0.29
Sex (male/female)	52/31	58/25	0.41
Duration of AF (months)	70±74	71±65	0.16
History of smoking	29	25	0.62
History of alcohol	15	21	0.35
Hypertension	40	37	0.76
Diabetes	11	10	NS
Hyperthyroidism	5	1	0.21
Stroke	7	13	0.23
Permanent pacemaker implantation	5	6	NS
Number of anticoagulant therapy			
Warfarin	28	34	0.42
Aspirin	38	34	0.64
Number of digitalis	28	27	NS
Number of AAD			
Beta receptor blocker	39	43	0.64
Amiodarone	35	37	0.88
LAD (mm)	53±11	51±12	0.25
LVED (mm)	48±5	48±6	0.92
LVEF (%)	61±7	62±9	0.25

AAD  = antiarrhythmic drugs; AF = atrial fibrillation; LAD  =  left atrial dimension; LVED  =  left ventricular end diastolic dimension; LVEF  =  left ventricular ejection fraction.

### 2.3 Catheter ablation

Ablation was guided by an electroanatomical mapping system (CARTO, Biosense Webster) and performed with a temperature-controlled, quadripolar, deflectable catheter with a 3.5-mm irrigated-tip catheter (Navistar, Biosense Webster) to encircle the left and right pulmonary vein (PV) 1 to 2 cm from their ostia, with additional lines in the posterior left atrium or roof and along the mitral isthmus. Two decapolar circular mapping catheters (Lasso, Biosense-Webster Inc) were placed inside the ipsilateral superior and inferior PVs or within the superior and inferior branches of a common PV to verify PVI during radiofrequency current ablation. RF current was delivered at each site until the local electrogram amplitude was reduced by at least 80%. Contiguous lesions were delivered at about 20 mm from the PV ostia. Additional ablation within the circles was performed in most of the patients, outside the PVs, where the local electrogram amplitude exceeded 0.2 mV. Additional lines were created in the posterior wall between the 2 encircling PV lesions and from the lower aspect of the left inferior PV to the mitral annulus. If AF was still present at the end of PVI, transthoracic cardioversion was used to restore sinus rhythm (SR). The end point was defined as the absence of any PV spike recorded on the 2 Lasso catheters placed within the ipsilateral PVs after at least 30 minutes following the PV isolation during SR.

### 2.4 Surgical Technique

**Figure 1:**
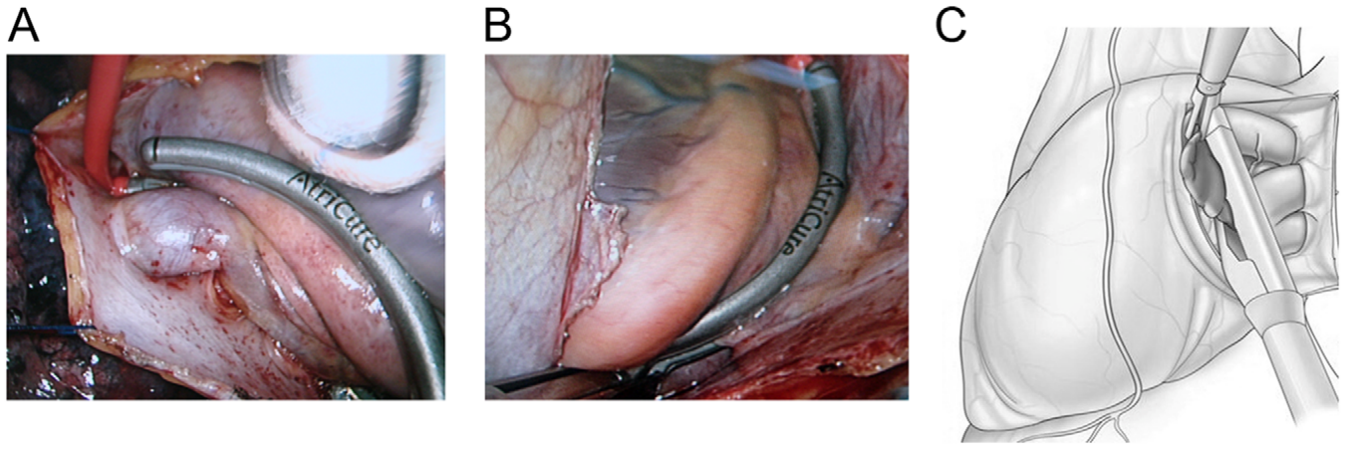
Video-assisted thoracoscopic surgical ablation. (A) Right thoracoscopic view of the bipolar clamp in place on the left atrial antrum, medial to the right PV. (B) Left thoracoscopic view of the bipolar clamp in place on the left atrial antrum, medial to the left PV. (C) The left atrial appendage is excised by stapling it with a stapler.

The video-assisted thoracoscopic surgical procedure was developed to perform an electrically PVI bilaterally. In brief a bipolar RF clamp and RF generator system (AtriCure, Inc, Cincinnati, Ohio) is used to achieve linear, transmural ablation lesions. The procedure is conducted after a general anesthesia administered with a double-lumen endotracheal tube and a transesophageal echocardiography used in the operating room is included in order to verify the left atrial appendage (LAA) excisions at the end of the procedures. As shown in Fig. 1 (A–B), each set of PV was encircled and ablated on the left atrium by using an epicardial-placed bipolar RF clamp and no other epicardial lesions were placed. The ligament of Marshall was taken down in all patients and a detailed ganglionic plexus (GP) mapping and ablation was performed. Thoracoscopic LAA exclusion was then performed using a Thoracic Endoscopic Linear Cutter EZ45G (Ethicon Endo-Surgery, Inc, Cincinnati, OH), with non buttressed 4.8-mm staples (Fig.1C). Appropriate stapler angle and adequate appendage coverage was enhanced by direct visualization through the working port.

### 2.5 Intraoperative Electrophysiologic Testing

Intraoperative electrophysiologic testing was performed, which includes bilateral PV antrum, baseline and post isolation sensing, pacing and GP detection. A baseline positive sensing result (rapid and disorderly atrial potentials) in the PV antrum area could be detected before PVI, and a negative sensing result (no atrial potentials) could be detected in the same area after ablation, which is called entrance block. A positive baseline pacing result is defined as the atrial and ventricular capture, what is the contraction of the atrium and ventricle in response to the electrical stimulus being sent from the temporary pacemaker (Oscor Pace 203H DDD External Dual-Chamber Pacemaker; Oscor Inc, Palm Harbor, Fla). A negative postablation pacing result means that no capture is obtained in the same area after ablation. A combined positive baseline pacing and negative postablation pacing result is called exit block. Achieving both entrance and exit block is regarded as a transmural lesion blocking of the conduction in the PV antrum area. Meanwhile, GP activity detection is included in the electrophysiologic testing procedure, as described by Mehall et al, which consist of a basically mapping by using a high-frequency stimulus (10 V, 800 times per second) for 5 seconds or more [8]. A positive response was defined as sinus bradycardia (<40 beats/min) or asystole, atrioventricular block, or hypotension occurring ventricular asystole of the onset of high-frequency stimulus.

### 2.6 Postablative Medical Management

Postablative anticoagulation treatment was done according to the American College of Cardiology/American Heart Association/European Society of Cardiology guidelines [9]. The decision whether to apply warfarin or aspirin has been done on the base of the patients Cardiac Failure, Hypertension, Age, Diabetes, Stroke (CHADS) score. Patients received 200 mg of amiodarone orally per day for three months. In the 4th month anticoagulant drug administration was discontinued when SR was present, what was examined by a 24–48 hour Holter monitoring. An AF or atrial flutter (AFL) episode was defined after documentation by means of ECG or Holter monitoring as at least 30 seconds lasting. During the follow-up period, if ECG analysis showed recurrence of AF or AFL which sustained for more than 8 hours and the anticoagulation value was adequately (International normalized ratio >2.0), a direct-current cardioversion was recommended, and a circumferential PV catheter ablation was performed.

### 2.7 Follow-up procedure

Follow-up controls were obtained from office visits at an outpatient building, mailed medical records received from local hospitals, and questionnaires. As a standard method for monitoring patient' heart rhythm we used a 24 to 48 hour Holter monitoring and additional twelve-lead ECG analysis for AF or AFL recurrence. Transthoracic UCG were evaluated at discharge and 1, 3, 6 and 12 months postoperatively. After one year, patients were seen every 6 months by their referring cardiologist. Free ECG examinations as well as free 24 to 48 hour Holter monitoring (Del Mar Reynolds Medical, Inc, Irvine, Calif) were offered at the Atrial Fibrillation Centre, Beijing Anzhen Hospital.

### 2.8 Ethical approval and consent

The protocol was approved by ( Institutional Review Board or Ethics Committee of Beijing Anzhen Hospital, Capital Medical University). All participants in this study gave a written informed consent.

### 2.9 Statistical analysis

Continuous data were presented as the mean ± standard deviation and categorical variables were expressed as the number of cases and percentage. The significance of the differences between the groups was assessed by the Student *t* test or Mann-Whitney *U* test for continuous variables and chi-square test for categorical variables. Cumulative event rates were calculated according to the Kaplan-Meier method. All tests were 2-tailed, and *p*<0.05 was considered significant. The data were analyzed with the SPSS for Windows version 12.0 (SPSS Inc., Chicago, IL, USA).

## Results

### 3.1 Immediate results during hospital observation

All patients successfully underwent the ablation procedure and there was no PV injury, in-hospital mortality, or necessity of reoperation because of bleeding. Circumferential PV catheter ablations were performed within a mean of 39±14 minutes of RF energy. The catheter group had a statistically longer procedure time for the entire ablation (231±27 minutes) than the surgical group for the entire operation (143.4±26.2 minutes) (*P* = 0.016). One patient with AF rhythm suffered from stroke in the surgical group, due to poor anticoagulation status (international normalized ratio<2.0 ), but recovered without any sequelae before discharge.

The changes in the cardiac rhythms during stay in hospital are described in Table 2. During ablation, AF termination (conversion in SR) was seen in 34.9% of the catheter group and in 45.7% of the surgical group of patients, respectively (*P* = 0.15). AF organization into AFL occurred more often in the catheter group, 24 patients (28.9%), 13 right sided and 11 left sided, than in the surgical group, 5 patients (6.0%), 2 right sided and 3 left sided (*P*<0.001). AF was documented to be 39.8% in total of the two groups. Temporary pacemakers were used in 4 patients from the surgical group because of bradycardia.

**Table 2:** Cardiac Rhythm in Hospital.

	Catheter GroupN = 83	Surgical GroupN = 83	p Value
Postoperative period			
SR	29(34.9)	38(45.7)	0.15
AF	30(36.2)	36(43.4)	0.39
AFL	24(28.9)	5(6.0)	<0.001
Pacemaker	0(0)	4(4.9)	0.12
Recurrence of AF/AFL			
AF	16 (19.3%)	12 (14.5%)	0.70
AFL	22 (26.5%)	2 (2.4%)	<0.001
At discharge			
SR	72(86.8)	53(63.9)	<0.001
AF	4(4.8)	24(28.9)	<0.001
AFL	6(7.2)	5(6.0)	NS
Pacemaker	1(1.2)	1(1.2)	NS

Data are presented as the number (%) of patients or mean ± SD. AF, atrial fibrillation; AFL, atrial flutter; SR, sinus rhythm.

During hospitalization, AF and AFL recurred in 16 (19.3%) and 22 (26.5%) of the patients in the catheter group after a mean time of 2.0±1.5 days, and 12 (14.5%) and 2 (2.4%) patients in the surgical group after a mean time of 6.0±1.4 days, respectively (*P* = 0.70 and *P*<0.001). At discharge from the hospital, SR and AF was maintained in 86.8% and 4.8% of the patients in the catheter group and 63.9% and 28.9% in the surgical group respectively (*P*<0.001).

The average length of stay in hospital was 6.1±2.6 and 4.8±2.5 days in the surgical group and catheter group, respectively (*P* = 0.56).

### 3.2 Follow-up Results

Average followed-up was 2.2years (range 1.0 to 3.6 years) after the procedures. During the follow-up period, two patients (2.4%) died in the catheter group, one for unknown reason and the other one with encephalorraghia. One patient (1.2%) died in the surgical group for unknown reason.

**Figure 2:**
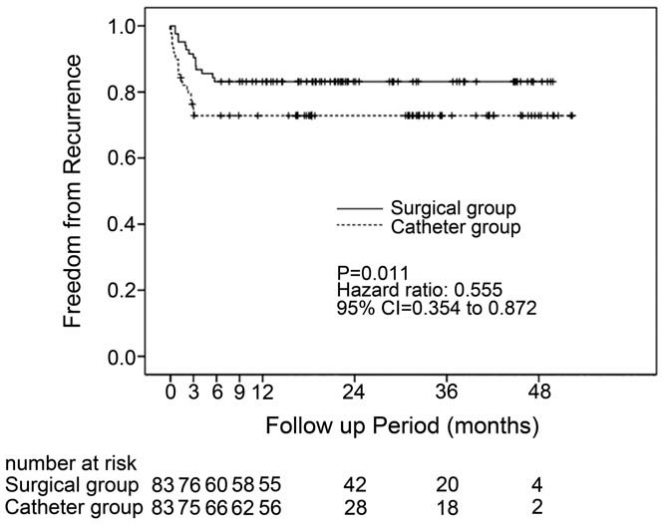
Freedom from recurrence after AF ablation. CI = confidence interval.

Taking a blanking period of 3 months after surgery into account, 49 (59.0%) and 62 patients (74.7%) did not have a ‘single’ registration of AF during follow-up in the catheter group and surgical group respectively (*P* = 0.047). Additionally, patients in the catheter group had a significantly higher rate of recurrent arrhythmia (*P* = 0.011, hazard ratio: 0.555, 95% CI: 0.354 to 0.872). (Fig.2)

In the catheter group, a second ablation procedure was performed in 23 patients with arrhythmia recurrence. Following the 2nd redo procedure, a third redo ablation was performed in 6 patients. Two patients who had failed the third redo ablation were treated then with the surgical procedure and were free from AF after follow-up.

Five patients with recurrent AF, within the surgical group, who agreed to a second treatment, underwent a catheter ablation successfully. Two of these patients were proven to have a bidirectional block based on tests performed during the surgery suffered recurrent AF. Subsequent CARTO electrophysiologic mapping showed complex fractionated atrial electrograms (CFAEs) in both atriums. The CFAEs ablation converted AF into atrial tachycardia, with the average AF cycle length of the 2 patients increasing from 140±12 ms to 214±23 ms after CFAEs ablation. Three-dimensional activation time-sequence mapping of the left atrium, combined with entrainment mapping, was then performed to clarify the underlying focal or macroreentry mechanism. The resulted “Entrainment” refers to higher tachycardia that occurs consistently during high frequency pacing; when the pacing frequency stops or is reduced below the original tachycardia, the tachycardia will drop back to a baseline level. The two patients were found to have several focal gaps originating from the roof and bottom of PVI lines. One of them a gap originating from the roof of the left superior PV down to the left inferior PV. In the third patient with atrial tachycardia a microreentrant cycle around the base of occlusive left appendage was found. An entrainment was performed to verify the reentrant mechanism. Interruption of the circuit as well as ablation of CFAEs mainly located at the posterior of the left atrial terminated the arrhythmia in this patient. The forth patient suffering AFL was identified anticlockwise (anterior of left atrial bottom area to posterior left atrial roof), with the average AF cycle length 302 ms. Ablation at the atrial roof between the two superior PVs, and ablation of CFAEs terminated the AFL. The fifth patients had mitral valve annulus-related atrial flutter. The creation of lesions from the left inferior PV to the mitral valve annulus was required to terminate the AFL in this patient. At the latest follow-up, all five patients were free of arrhythmias and independent of antiarrhythmic drug (AAD).

Frequently, the decision to terminate AAD was made after 3-month monitoring. For patient who had a successful procedure, the freedom from AAD was 44.6% and 61.4% in catheter group and surgical group respectively (*P* = 0.043).

During the follow-up, cerebrovascular accidents occurred in two patients (2.4%) of the surgical group, and in one patient (1.2%) of the catheter group. The 1- and 3- year actuarial survival free from stroke rates were both 99.0%±1.0% in the catheter group, and both 96.0%±1.5% in the surgical group, respectively (*P* = 0.27).

Eleven (6.6%) patients had to undergo preoperative permanent pacemaker implantation because of sick SR. During the follow-up, in three patients (3.6%) permanent pacemakers were implanted in the catheter group and in one patient (1.2%) of the surgical group. The 1- and 3- year actuarial survival free from implanting permanent pacemaker were both 97.0%±2.0% in the catheter group, and 100% and 97.0±3.0% in surgical group, respectively (*P* = 0.20).

## Discussion

### 4.1 Main findings

This is the first case-matched retrospective study comparing catheter and video-assisted minimally invasive ablation techniques in patients with long-standing persistent AF. Although at discharge from the hospital, the maintenance of SR was higher in the catheter group, we found that minimally invasive ablation resulted in long-term maintenance of SR in 74.7 percent of the patients (vs. 59.0% in the catheter group, *P* = 0.047). Additionally, a Kaplan-Meier analysis showed that patients in the catheter group had a significantly higher rate of recurrent arrhythmia.

### 4.2 Catheter ablation

Catheter-based PVI is relatively effective in maintaining SR for paroxysmal AF (65% to 70% success rate); for non paroxysmal AF, however, the PVI success rate is dramatically lower (20% to 30%) [10]–[12]. Therefore, different strategies adjunctive to PVI or using other concepts have been developed. These strategies include: creation of various ablation lines (mitral isthmus, roof line, posterior lines to isolate the posterior wall); ablation based on the CFAEs as well as cardiac autonomic denervation [13]–[16]. Oral et al evaluated catheter ablation with chronic AF. After a follow-up period of 7–14 months, only a mean of 33% of patients were in SR [17]. Tilz et al reported on 205 consecutive patients with long-standing persistent AF who underwent PVI, left linear and CFAEs catheter ablation. After a mean of 1.7±0.8 procedures, 135 of 199 patients (67.8%) remained in SR. Eighty-six patients (43.2%) remained in SR following PVI performed as the sole ablative strategy [16] and our results are in accordance with these previous reports who also adopted this technique. The PVI procedure appears to have a low success rate; however, the comparative advantages of a minimally invasive procedure have not been systematically evaluated yet.

### 4.3 Minimally invasive ablation

The success rate achieved in the most challenging surgical groups of patients, those with long-standing persistent AF, was not clear. Earlier in our series, the long-standing persistent AF group shows 44.4% and 71.4% SR restoration rates at 3 and 6 months postoperatively, respectively [18]. Similar research also reports 67% and 72% success rates, respectively [19], [20].

Meanwhile our results show an optimistic therapeutic effect for long-standing persistent AF. This could be either due to a successful addition of bipolar ablation or due to the additional GP-ablation. During the minimally invasive operation, the energy is delivered between the jaws together with a transmurality feedback algorithm, and the transmurality of the isolation lesion is theoretically ensured. Besides the transmurality algorithm and facilitated multiple isolation lesions, the epicardial electrophysiologic testing further helps to evaluate the quality of the atrial lesion. What needs to be specially mentioned is that there are possible (24 patients in the surgical group (28.9%)) persistent regular and small potentials sensed even if ablations were done on a certain side. Often, these diminutive potentials recorded after ablation can represent “far-field” left atrial potentials. The effect of this “positive” postablation sensing and GP detection results on short-term and midterm therapeutic outcomes is under observation in a concurrent study. In this study pacing results show that all patients who were in SR after bilateral ablations did not show “atrial and ventricular capture” and accordingly achieved “exit block”.

Investigators have showed that conversion of a premature depolarisation to AF depends on GP stimulation [21]. They could even initiate ectopic foci from the PV by GP stimulation alone. It is clear that the autonomic nervous system plays a key role in the initiation and maintenance of AF. Pappone first showed that patients with a vagal response during ablation had a higher success rate in comparison to patients without a vagal response [22]. Scherlag were the first to compare PVI with and without GP-ablation in a prospective study [23]. The follow-up data suggest better outcome with additional GP-ablation. The reported success rates vary between 29% and 84% [24]. Earlier in our series, ectopic foci outside the PV played an important role in persistent AF [19]. GP-ablation is, therefore, expected to contribute to the success of procedures for long-standing persistent AF.This study supports the feasibility of amputation of the LAA through the minimally invasive technique. In addition after amputation of the LAA, postoperative thromboembolic events were substantially reduced. During the follow-up, only two patients had cerebrovascular events.

### 4.4 Study limitations

The main limitation of this study is the retrospectivity. A larger, randomized and prospective trial with catheter based and minimally invasive ablation for lone AF is underway and will better define the efficacy of these two procedures in accordance with published guidelines.

A second limitation is that PVI with GP-ablation is not adequate to treat long-standing persistent AF. Patients with long-standing persistent AF pose a challenge because various pathophysiologic changes that occur in the atria of such patients may adversely affect the success of various treatment strategies. We reported electrophysiologic findings in patients presenting with recurrent atrial arrhythmia after minimally invasive procedures. Gaps at the roof and bottom of the PVI ring may contribute to the underlying mechanism of recurrent arrhythmias [18]. The connecting lesions between the left superior PV and left appendage and right inferior PV to the mitral valve annulus, as well as the roof line of the LA are all important; however, it is difficult to ablate these sites when the heart is beating using the present device during minimally invasive procedures. With the recently available Coolrail (AtriCure, Inc, West Chester, Ohio, USA), totally left-sided maze III lesion sets can be achieved relatively easy. In long-standing persistent AF, the efficacy of this procedure could benefit from its more extensive lesion set.

Our results suggest that in patients with long-standing persistent AF, the minimally invasive PVI with GP-ablation had the better ability to maintain SR. A further randomized study assessing the role of minimally invasive surgery in AF patients seems to be justified.
